# Role of the microbiota–gut–lung axis in the pathogenesis of pulmonary disease in children and novel therapeutic strategies

**DOI:** 10.3389/fimmu.2025.1636876

**Published:** 2025-09-25

**Authors:** Zhifang Wang, Jiayao Yu, Yaqi Liu, Jiaqi Gong, Zepeng Hu, Zheng Liu

**Affiliations:** ^1^ Department of Pediatrics, Shaoxing Keqiao Women and Children’s Hospital, Shaoxing, Zhejiang, China; ^2^ Department of Medical Imaging, School of Medicine, Shaoxing University, Shaoxing, Zhejiang, China; ^3^ Department of Nursing, School of Medicine, Shaoxing University, Shaoxing, Zhejiang, China; ^4^ Department of Clinical Medicine, School of Medicine, Shaoxing University, Shaoxing, Zhejiang, China; ^5^ Department of Pharmacology, School of Medicine, Shaoxing University, Shaoxing, Zhejiang, China

**Keywords:** gut–lung axis, pediatric respiratory diseases, microbial metabolites, multi-omics, precision medicine

## Abstract

Emerging evidence highlights the microbiota–gut–lung axis (MGLA) as a pivotal regulator of pediatric respiratory health, yet mechanistic insights are lacking and therapeutic applications remain unclear. This review synthesizes cutting-edge findings to delineate how gut microbiota-derived metabolites, particularly short-chain fatty acids (SCFAs), orchestrate pulmonary immunity and disease pathogenesis in children. Leveraging multi-omics integration (metagenomics, metabolomics, transcriptomics), emerging studies have uncovered novel microbe–host interactions driving immune dysregulation in asthma, pneumonia, and cystic fibrosis. A comprehensive map of gut–lung crosstalk has been established across these conditions. Current studies suggest that early-life gut dysbiosis, shaped by delivery mode, antibiotics, and diet, disrupts SCFA-mediated immune homeostasis, amplifying T-helper 2 cell inflammation and impairing alveolar macrophage function. Crucially, we identified disease-specific microbial signatures (e.g., depletion of *Lachnospira* and *Faecalibacterium* in asthma) and demonstrated that fecal microbiota transplantation and probiotic interventions restore microbial balance, attenuating airway inflammation in preclinical models. This work pioneers the translation of MGLA insights into precision medicine strategies, highlighting dietary modulation and microbial therapeutics as viable alternatives to conventional treatments. By bridging microbial ecology and immune dynamics, our findings provide actionable biomarkers for early diagnosis and personalized interventions, addressing critical gaps in pediatric respiratory disease management. The integration of multi-omics frameworks not only advances mechanistic understanding but also positions the MGLA as a transformative target in reducing global childhood morbidity. Future research must prioritize longitudinal studies and clinical trials to validate these innovations, ultimately redefining therapeutic paradigms for GLA-driven pathologies.

## Graphical Abstract

Regulatory Mechanisms and Therapeutic Strategies of the GLA in Pediatric Pulmonary Diseases. The gut microbiota interacts bidirectionally with the pulmonary microenvironment through microbial metabolites (e.g., SCFAs) and immune signaling molecules (e.g., IFN, IL-6, IL-10), forming a central mechanism underlying this axis. Gut dysbiosis disrupts SCFA-mediated immune homeostasis, contributing to the pathogenesis of childhood respiratory diseases such as asthma, pneumonia, and cystic fibrosis. Integrated multi-omics studies have identified disease-specific microbial signatures and propose novel strategies—including FMT, probiotics, prebiotics, and dietary interventions—to restore gut microbiota equilibrium, suppress airway inflammation, and advance precision-based therapeutic approaches for pediatric respiratory disorders. CF, cystic fibrosis; FMT, fecal microbiota transplantation; GLA, gut–lung axis; IFN, interferon; IL, interleukin; OSAS, obstructive sleep apnea syndrome; SCFAs, short-chain fatty acids.

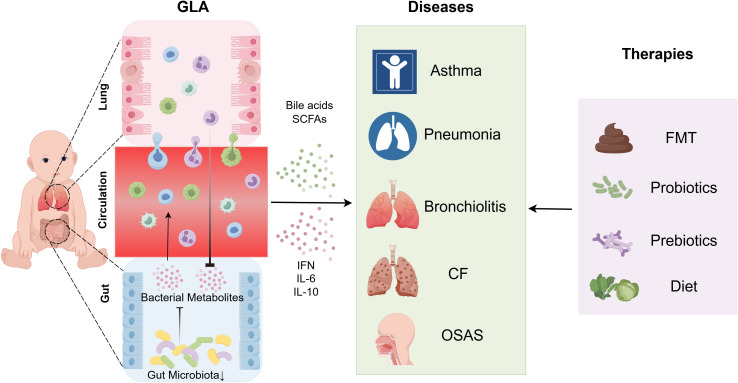

## 1 Introduction

Acute respiratory infections and influenza-like illnesses (ILIs) account for approximately 10% of annual outpatient visits globally, with pediatric populations bearing a disproportionate burden. Children aged 1–17 years exhibit a fourfold higher incidence of ILIs than adults (1). This vulnerability stems from structurally immature airways and dynamically evolving immune regulation, creating a suitable environment for recurrent infections. Beyond acute morbidity, such episodes disrupt developmental homeostasis and amplify lifelong cardiopulmonary risks (2), necessitating urgent research into early-life susceptibility mechanisms.

The human microbiome associated with the respiratory tract is characterized by its diversity, heterogeneity, and dynamism. The complexity of the microbiome, along with the intricate interactions among microorganisms, host cells, and the host immune system, involves multiple factors. There is often an interaction between gut and respiratory microbiota, with the lymphatic system providing a direct pathway, known as the gut–lung axis (GLA), via which the gut microbiome can influence outcomes related to respiratory diseases and modulate the host’s immune response (1). The GLA is a bidirectional communication network mediated by microbiota and metabolites that links intestinal and pulmonary health (3). Emerging evidence indicates that perturbations in one organ (e.g., gut dysbiosis (4)) can propagate systemic immune dysregulation, exacerbating pathologies in the other (e.g., asthma exacerbations (5)). For example, gut-derived microbial components such as lipopolysaccharide (LPS) modulate pulmonary inflammation via lymphatic dissemination (6) whereas respiratory viral infections reciprocally reshape the gut microbial ecology (7). Despite these advances, the spatiotemporal dynamics of microbiome–immune crosstalk in pediatric populations remain poorly mapped.

The gut microbiome critically regulates pediatric health, with its assembly during infancy dictating immune maturation trajectories (8). The composition of the intestinal microbiome influences health from before birth into childhood, with numerous diseases linked to imbalances in this ecosystem. The gut microbiome evolves continuously from infancy to maturity, influenced by various factors that shape its development and composition. Characteristics of the gut microbiota can impact brain development, immune function, lung health, and overall physical growth (9). Gut microbial dysbiosis can have long-term consequences, as supported by data from mouse models in which mice have an increased predisposition to allergic inflammation following early-life antibiotic use (10, 11). Early-life disruptions, such as antibiotic exposure or dietary insufficiency, induce persistent dysbiosis, elevating the risks of allergic sensitization and recurrent respiratory infections (12).

Microbial metabolites such as short-chain fatty acids (SCFAs) emerge as key orchestrators of mucosal immunity, yet their role in gut–lung interactions remains underexplored in children. Recent studies have integrated multi-omics approaches (metagenomics, metabolomics, transcriptomics) to decode microbiota–immune networks in pediatric respiratory diseases. These efforts have identified disease-specific microbial signatures (e.g., *Lachnospira* depletion in asthma) and have demonstrated that SCFAs enhance alveolar macrophage function not only via epigenetic modulation but also by inducing tolerance-related genetic signatures (13). Such findings highlight the potential of microbiota-targeted therapies (e.g., probiotics, dietary interventions) to recalibrate immune homeostasis.

## 2 Gut microbiota and children’s respiratory health

### 2.1 Characteristics of the pediatric gut and respiratory microbiota

The pediatric gut microbiome represents a dynamic ecosystem of bacteria, fungi, viruses, and archaea. Indeed, the human gut is inhabited by between 100 thousand and 100 billion bacteria per milliliter of luminal content depending on the region and is therefore the most densely colonized organ (14). Crucially, this microbial consortium not only supports nutrient metabolism but also calibrates systemic immunity via endocrine and metabolic crosstalk (15, 16). This symbiotic relationship is established from birth with the infant gut microbiota and continues to evolve during the critical early years of life. During this period, infants experience rapid growth, with substantial increases in height, weight, and head circumference. Concurrently, their metabolic organs, immune system, digestive system, and neurocognitive functions undergo substantial development and maturation. This phase is also pivotal for formation of the gut microbiota, which is essential for maintaining overall health (17).

In contrast to the gut, the respiratory microbiome exhibits spatiotemporal stratification—nasal cavities favor *Staphylococcus and Corynebacterium*, whereas lower airways are dominated by Prevotella and Streptococcus—with bacterial loads (10^3–^10^5^ CFU/mg) that are orders of magnitude lower than intestinal levels (18, 19). Our understanding of bacterial component development in the airways is more limited than our knowledge of the gut microbiota. Some evidence (primarily from mouse models) suggests that the respiratory microbiota matures during childhood, a process that is crucial for promoting tolerance to airborne allergens (20). Despite shared dominant phyla (Bacteroidetes/Firmicutes), the lung microbiome displays unique assembly rules; microbial immigration (inhalation/microaspiration), elimination (mucociliary clearance), and local replication dynamically sculpt the community structure (21).

Deciphering the co-evolution of gut and respiratory microbiomes is fundamental to understanding pediatric immune ontogeny. During early life, these microbial communities transcend passive colonization; they actively direct immune cell differentiation via metabolite signaling (e.g., SCFAs), epigenetic modulation, and pathogen exclusion (22). Integrated analysis of human cohort data has revealed that synchronized gut–lung microbiota maturation establishes a systemic immune “set-point,” dysregulation of which underlies susceptibility to pneumonia and asthma. This paradigm shifts the focus from cataloging microbial taxa to decoding their functional networks, a cornerstone of our therapeutic discovery platform.

### 2.2 Gut microbiota affects development of the immune system in children

Upon birth, infants start to develop their initial microbiome, with its composition being shaped by the delivery method, whether through natural, vaginal, or cesarean birth (23). The initial bacterial colonization and various other factors following childbirth strongly influence an infant’s early-life microbiome, which subsequently regulates immune system development of the newborn (24). Whereas vaginal birth establishes a maternally derived microbiome enriched in *Lactobacillus* and *Bifidobacterium*, cesarean delivery favors skin-associated *Staphylococcus* and environmental taxa (25). Crucially, this immunomodulatory process exhibits developmental stage-specific sensitivity, as evidenced by murine and human studies demonstrating irreversible immune programming defects when microbial exposure is disrupted during early postnatal windows (26, 27). This foundational microbial assembly orchestrates lymphoid tissue development and immune cell education—processes that are vulnerable to disruption by antibiotics or formula feeding. Such dysbiosis propagates systemic immune misprogramming, elevating the risks for asthma and obesity via GLA signaling (28).

The gut microbiome operates as a microbial tutor during infancy, instructing immune cell differentiation through metabolite- and antigen-driven dialogues. Key taxa (e.g., Clostridia clusters) promote expansion of regulatory T cells (Tregs) via SCFA production, and segmented filamentous bacteria drive T-helper 17 cell (Th17) polarization via interleukin 23 (IL-23)/IL-17 axis activation (29). This trans-organ immunity, mediated by circulating microbial metabolites and trained immune cells, forms the mechanistic bedrock of the GLA (30).

In the intestinal environment, pattern recognition receptors (PRRs), including toll-like receptors (TLRs), C-type lectin receptors, NOD-like receptors, and RIG-I-like receptors, can identify specific molecular patterns in the microbiota, thereby initiating immune responses. The activation of PRRs not only promotes the maturation and activation of immune cells but also modulates their migration and function through the production of cytokines and chemokines (31). Specifically activated PRRs can induce dendritic cells (DCs) to produce IL-12p70, a crucial Th1-polarizing cytokine for anti-infection and anti-tumor immune responses. Moreover, PRR activation enhances the expression of surface molecules such as CD80, CD86, and human leukocyte antigen-DR (HLA-DR) on DCs, thereby improving their antigen-presenting capacity and T-cell activation ability (32).

**Figure 1:**
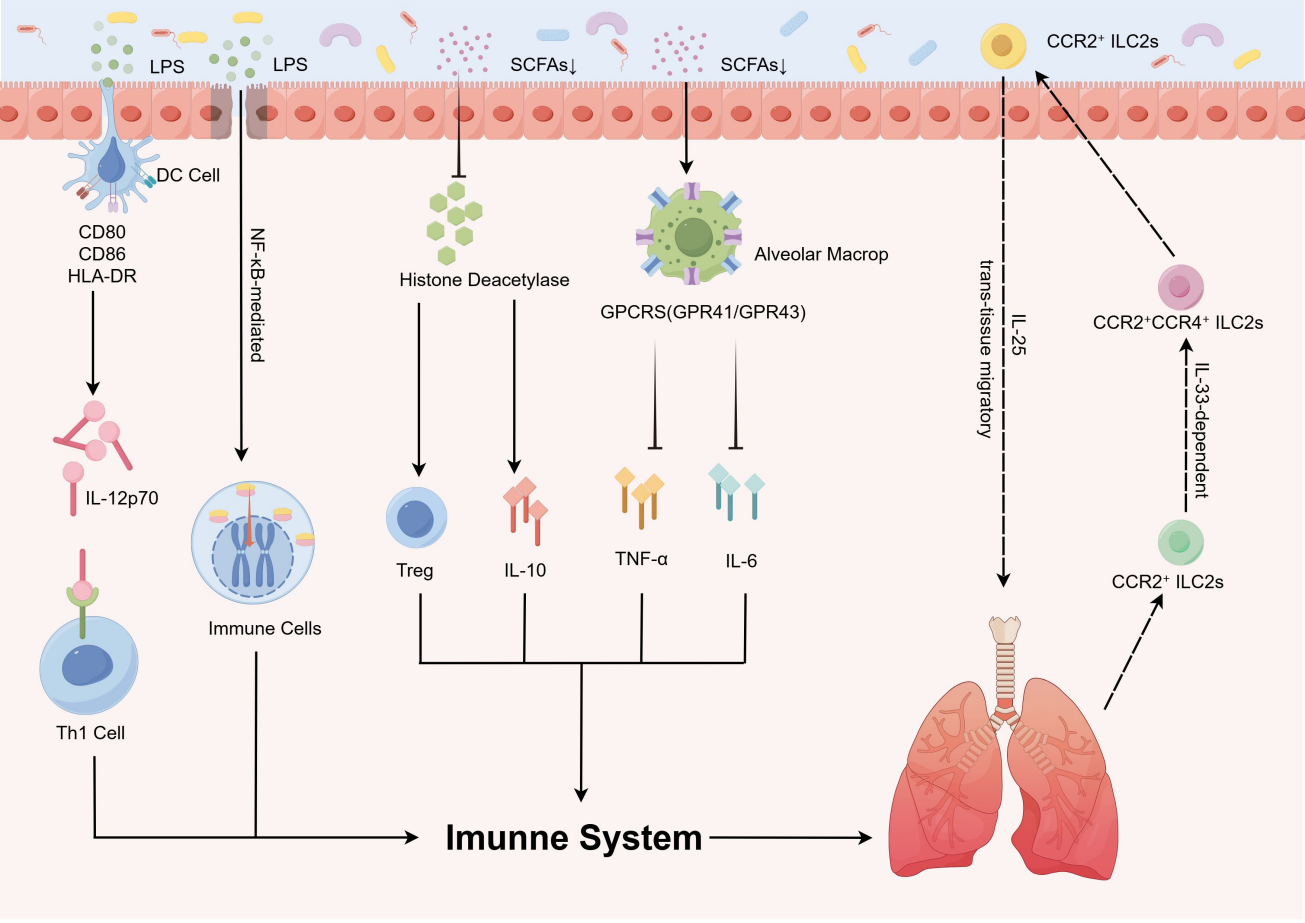
Gut microbiota modulates pathophysiological processes in pediatric pulmonary diseases via immune regulatory mechanisms. The gut microbiota orchestrates immune regulatory mechanisms that influence the pathogenesis of childhood pulmonary diseases via microbial metabolites (e.g., SCFAs and LPS) and immune signaling networks, which critically regulate immune system development and disease progression. DCs recognize LPS, leading to upregulated CD80/CD86/HLA-DR expression, enhanced antigen-presenting capacity, and IL-12p70 secretion, thereby driving Th1 polarization to reinforce anti-infective immunity. Gut-derived LPS activates the TLR4/NF-κB pathway, triggering pulmonary inflammation. SCFAs inhibit histone deacetylase activity to promote Treg differentiation and anti-inflammatory IL-10 secretion, while simultaneously activating alveolar macrophages via GPCRs to suppress pro-inflammatory cytokine (TNF-α, IL-6) release. Additionally, trans-tissue migration of CCR2^+^ ILC2s further modulates pediatric pulmonary disease pathogenesis. CCR2+CCR4+ILC2s, C-C chemokine receptor type 2-positive and C-C chemokine receptor type 4-positive type 2 innate lymphoid cells; CCR2+ILC2s, C-C chemokine receptor type 2-positive type 2 innate lymphoid cells; CD80/CD86, cluster of differentiation 80/86; DC, dendritic cell; GPCRs (GPR41/GPR43), G protein-coupled receptors (G protein-coupled receptor 41/G protein-coupled receptor 43); HLA-DR, human leukocyte antigen – DR isotype; IL, interleukin; LPS, lipopolysaccharide; SCFAs, short-chain fatty acids; Th1, T helper 1 cells; TNF-α, tumor necrosis factor alpha; Treg, regulatory T cells.

A previous study redefined the gut microbiome as a systemic immune rheostat, with microbial metabolites (e.g., SCFAs, indoles) serving as endocrine-like messengers that synchronize lung immunity (33). Via integrated metagenomic–metabolomic analysis, spatial gradients of these molecules have been mapped from gut to bronchoalveolar lavage, correlating their depletion with neutrophilic inflammation in severe asthma (34) (**Figure 1**). This paradigm-shifting discovery positions fecal metabolite profiling as a noninvasive biomarker for predicting corticosteroid responsiveness—a cornerstone of our precision pulmonology framework. By decoding the gut–lung dialogue, we can pioneer microbiota-centric strategies to reset immune homeostasis in pediatric respiratory diseases.

## 3 Role of the GLA in children’s pulmonary physiological function

### 3.1 Core immunoregulatory mechanisms of the GLA

The GLA operates as a bidirectional immunoregulatory circuit wherein microbial components and metabolites systemically modulate pulmonary immunity via three primary mechanisms: 1) molecular mimicry: bacterial LPS primes alveolar macrophages via TLR4 (35); 2) metabolite trafficking: gut-derived SCFAs, particularly propionate and butyrate, suppress IL-13-driven eosinophilia by enhancing airway epithelial barrier integrity (36); 3) immune-cell migration: intestinal Th17 cells recruited to the lungs exacerbate neutrophilic inflammation in asthma, a process amplified by dysbiosis-induced IL-23 signaling (37). Disruption of gut barrier integrity promotes microbial translocation (e.g., circulating LPS), which triggers nuclear factor-kappa B (NF-κB)-mediated pulmonary hyperinflammation (38). A murine study demonstrated that elevated circulating propionate levels promote hematopoiesis of DC precursors in bone marrow, subsequently affecting DCs in the lungs and draining lymph nodes, thereby resulting in an impaired ability of these DCs to activate Th2 cells within the lung tissue (39). These pathways collectively enhance intestinal mucosal immunity while systemically modulating immune cell differentiation and function. Notably, animal studies substantiate that SCFAs can attenuate pulmonary inflammation by activating Tregs and conditioning DCs to suppress Th2-polarized responses in murine models, thereby recalibrating Th1/Th2 equilibrium (40) (**Figure 1**, **Table 1**).

**Table 1:** Multi-omic insights into gut–lung axis mechanisms in respiratory diseases.

Omics technology	Study participants/disease	Key findings	Mechanism/functional relevance	Ref.
Metagenomics	Children with asthma	Reduced abundance of *Lachnospira*, *Veillonella*, and *Faecalibacterium* in the gut microbiota of 3-month-old infants	Reduced microbial diversity correlates with heightened asthma risk	(52) (53)
	Pneumonia (mouse model)	Gut microbiota dysbiosis (*Bifidobacterium*↓) → impaired phagocytic function of alveolar macrophages	Acetate enhances anti-*Streptococcus pneumoniae* capacity via GPR43 signaling	(64)
	Patients with cystic fibrosis	Depletion of *Bacteroides* and *Faecalibacterium prausnitzii* in the gut	Propionate deficiency impairs neutrophil function; bile acid dysregulation exacerbates airway inflammation	(81) (86) (87)
Metabolomics	Asthma (mouse model)	Decreased fecal SCFA levels (propionate, butyrate)	SCFA depletion promotes Th2 inflammation via HDAC-dependent pathways	(11) (40)
	Patients with COVID-19	Impaired L-isoleucine biosynthesis and reduced acetate levels	Metabolic dysregulation drives immune dysfunction and intestinal barrier disruption	(98)
	OSAS (children)	Reduced butyrate levels → compromised intestinal barrier integrity	SCFA deficiency exacerbates systemic inflammation and hypertension risk	(95)
Transcriptomics	RSV bronchiolitis (mouse model)	Acetate activates GPR43 → NF-κB pathway → enhanced IFN-β production	Augmented antiviral immunity attenuates RSV severity	(77)
	Patients with asthma (alveolar macrophages)	SCFAs (butyrate) inhibit HDAC activity	SCFAs promote Treg differentiation via epigenetic modulation	(42) (44)
	Pneumonia (mouse macrophage model)	Gut microbiota depletion → metabolic reprogramming in alveolar macrophages	Microbial metabolites (e.g., acetate) regulate macrophage function through GPR43 signaling	(65)

GPR43, G protein-coupled receptor 43; HDAC, histone deacetylase; IFN-β, interferon-beta; NF-κB, nuclear factor kappa-light-chain-enhancer of activated B cells; OSAS, obstructive sleep apnea syndrome; RSV, respiratory syncytial virus; SCFA, short-chain fatty acids; Th2, T helper type 2; Treg, regulatory T cells.

### 3.2 Systemic immunomodulatory roles of SCFAs

SCFAs—primarily acetate, propionate, and butyrate—serve as keystone metabolites linking the gut microbiota to pulmonary immune homeostasis (11, 41). Emerging evidence suggests that elevated levels of SCFAs in infants are associated with a reduced incidence of asthma, potentially mediated through dual anti-inflammatory mechanisms (42). First, SCFAs activate G protein-coupled receptors (GPR41/GPR43) on alveolar macrophages via receptor-dependent signaling, enhancing pathogen clearance while suppressing pro-inflammatory cytokine release (e.g., tumor necrosis factor alpha [TNF-α], IL-6) (22, 43). Second, SCFAs modulate immune cell function through epigenetic regulation: by inhibiting histone deacetylase (HDAC) activity; these promote anti-inflammatory factor expression (including IL-10) and Treg differentiation while simultaneously restraining Th17 cell overactivation. These coordinated actions maintain pulmonary immune homeostasis and mitigate inflammation (42, 44) (**Table 1**). Although SCFA concentrations are low in the lungs, gut-derived propionate can induce bone marrow precursors to generate macrophages. These macrophages then migrate to the lungs and attenuate allergic inflammation by promoting IL-10 production (11). These findings establish SCFAs as systemic immunomodulators that exert hormone-like signaling functions (45). Collectively, the above findings underscore SCFAs as pivotal gut-derived mediators that orchestrate pulmonary immune homeostasis via receptor-dependent signaling and epigenetic mechanisms. The ability of SCFAs to prime anti-inflammatory responses both locally and systemically highlights their therapeutic potential in asthma and other inflammatory lung diseases (**Figure 1**).

### 3.3 Trans-organ migration mechanisms of immune cells

Emerging evidence highlights the pivotal role of immune cell trafficking in gut–lung communication wherein innate lymphoid cells (ILCs) and gut-primed Tregs migrate to pulmonary tissues via mesenteric lymphatics under chemokine guidance (46). Extending these observations, one study provided mechanistic insights into the cytokine-regulated bidirectional trafficking of ILC2s using integrated pseudotemporal analysis, lineage tracing, and ectopic transplantation assays. That study delineated a unidirectional maturation trajectory wherein lung-derived C-C chemokine receptor type 2-positive (CCR2^+^) ILC2s migrate to the intestine via IL-33-dependent pathways, undergoing phenotypic transition characterized by a transient CCR2^+^CCR4^+^ double-positive population and terminal differentiation into CCR4^+^ gut-resident ILC2s. This migratory axis demonstrates strict tissue tropism under homeostasis, with no detectable reverse migration from intestinal ILC2s to pulmonary niches. Strikingly, IL-25 stimulation subverts this physiological unidirectionality, potentiating intestinal ILC2s to acquire trans-tissue migratory competence toward pulmonary compartments (47). This cellular crosstalk establishes a “mobile immune network” that integrates microbial signals and tissue-specific immunity (**Figure 1**).

### 3.4 Translational implications and future directions

The GLA, which is crucial for immune balance and respiratory health, strongly influences pediatric pulmonary diseases. Understanding its role in diseases such as asthma and bronchopulmonary dysplasia could offer new therapeutic strategies. Future research priorities include standardizing methodologies for gut microbiota and metabolite profiling in pediatric cohorts, elucidating age-dependent variations in GLA functionality, and developing targeted interventions (e.g., SCFA prodrugs, microbiota transplantation) to restore immune homeostasis.

## 4 Role of the GLA in pediatric pulmonary diseases

### 4.1 Asthma

**Figure 2:**
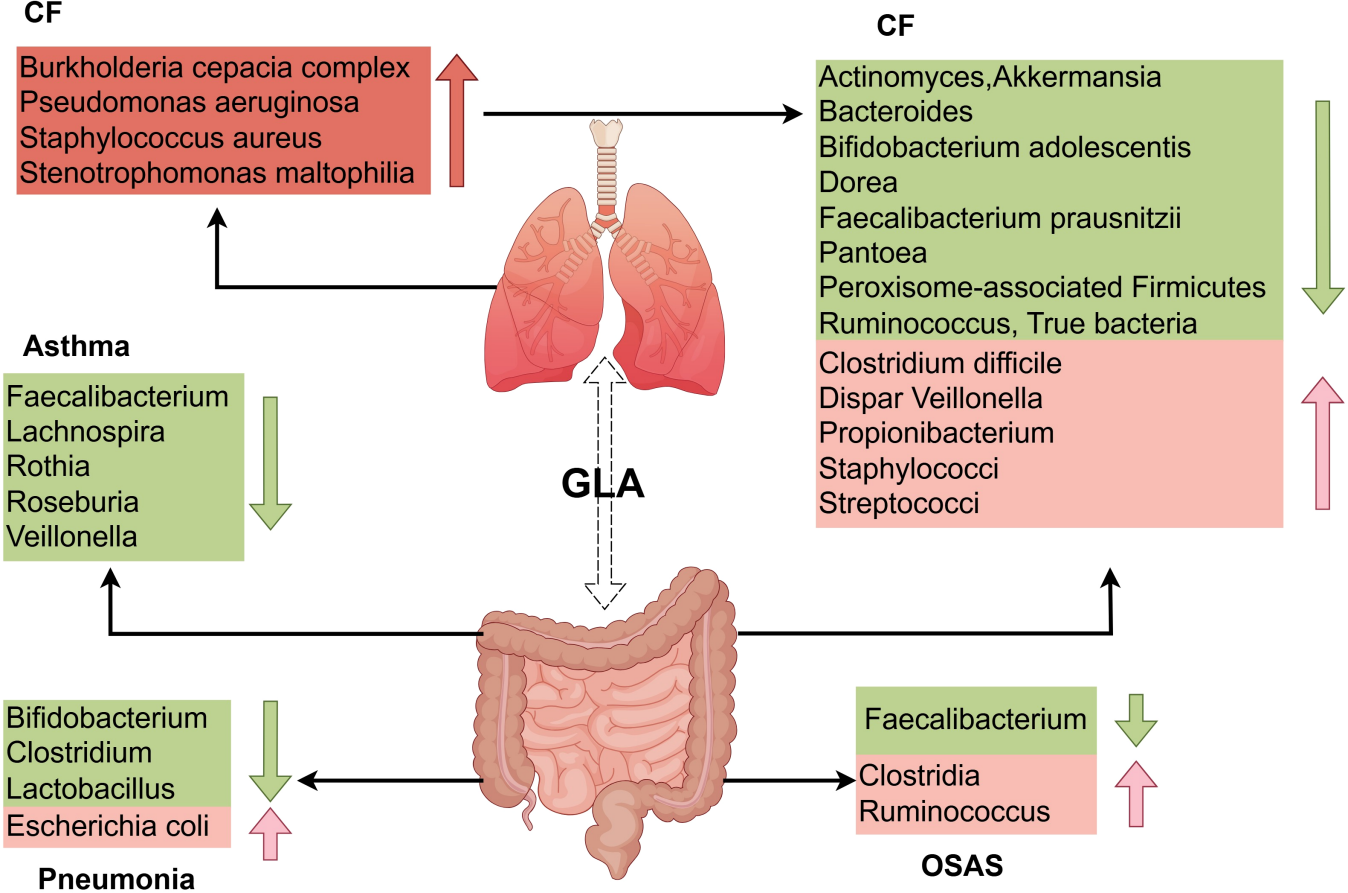
Distinct features of gut and respiratory microbiota dysbiosis in pediatric pulmonary diseases. The GLA exhibits dynamic microbiota alterations in childhood asthma, pneumonia, CF, and OSAS. Children with asthma have reduced gut abundance of immunomodulatory taxa (e.g., *Lachnospira, Faecalibacterium, Rothia*) alongside expansion of pro-inflammatory genera (e.g., Enterococcus). Patients with pneumonia display diminished intestinal Bifidobacterium and Lactobacillus abundance with concurrent enrichment of *Escherichia coli*. In CF, the gut microbiota is characterized by substantial enrichment of opportunistic pathogens (e.g., Burkholderia cepacia complex, Pseudomonas aeruginosa) and depletion of beneficial species (e.g., Bacteroides, Faecalibacterium prausnitzii). OSAS is associated with reduced levels of SCFA-producing bacteria (e.g., Clostridia, Ruminococcus), which exacerbates systemic inflammation via the metabolic–immune axis. GLA, gut–lung axis; CF, cystic fibrosis; OSAS, obstructive sleep apnea syndrome; SCFAs, short-chain fatty acids.

Asthma remains a leading chronic pediatric condition, accounting for approximately 400,000 annual deaths globally (48, 49). The current global prevalence of asthma exceeds 300 million individuals, with a projected increase to 400 million by 2025 (50). Emerging evidence implicates GLA dysbiosis in asthma pathogenesis, with microbial alterations influenced by perinatal factors including cesarean delivery, neonatal antibiotic exposure, maternal nutritional patterns, formula supplementation, and microbial environmental exposures (51, 52). Longitudinal cohort studies demonstrate that diminished gut microbial diversity during early infancy correlates with subsequent asthma development. Infants who develop asthma exhibit significantly reduced α-diversity metrics as early as 1 month postpartum (52), with temporal microbiota dynamics showing critical developmental windows for asthma predisposition. Specifically, transient depletion of immunomodulatory taxa (*Lachnospira*, *Veillonella*, Faecalibacterium, and *Rothia*) at 3 months postnatally distinguishes high-risk infants (53) (**Table 1**). Phylum-level analysis reveals Firmicutes dysregulation in pediatric patients with asthma, characterized by decreased anti-inflammatory species (e.g., *Roseburia*) and expansion of pro-inflammatory genera (*Enterococcus*, *Clostridium*) (54) (**Figure 2**).

This ecological imbalance coincides with impaired mucosal immune programming during critical developmental windows, as evidenced by multi-omics integration of microbial community dynamics, host metabolomic signatures, and nutritional patterns (49, 55, 56). Notably, gut dysbiosis, particularly the depletion of *Akkermansia*, *Bifidobacterium*, and *Faecalibacterium* species during critical developmental windows, is strongly associated with heightened susceptibility to asthma and allergic disorders (26, 27) (**Figure 2**). Mechanistically, microbial metabolic output—particularly SCFA biosynthesis—mediates dietary microbiota–immune crosstalk, with reduced SCFA production correlating with asthma risk in low-diversity microbiomes (1, 11).

**Figure 3:**
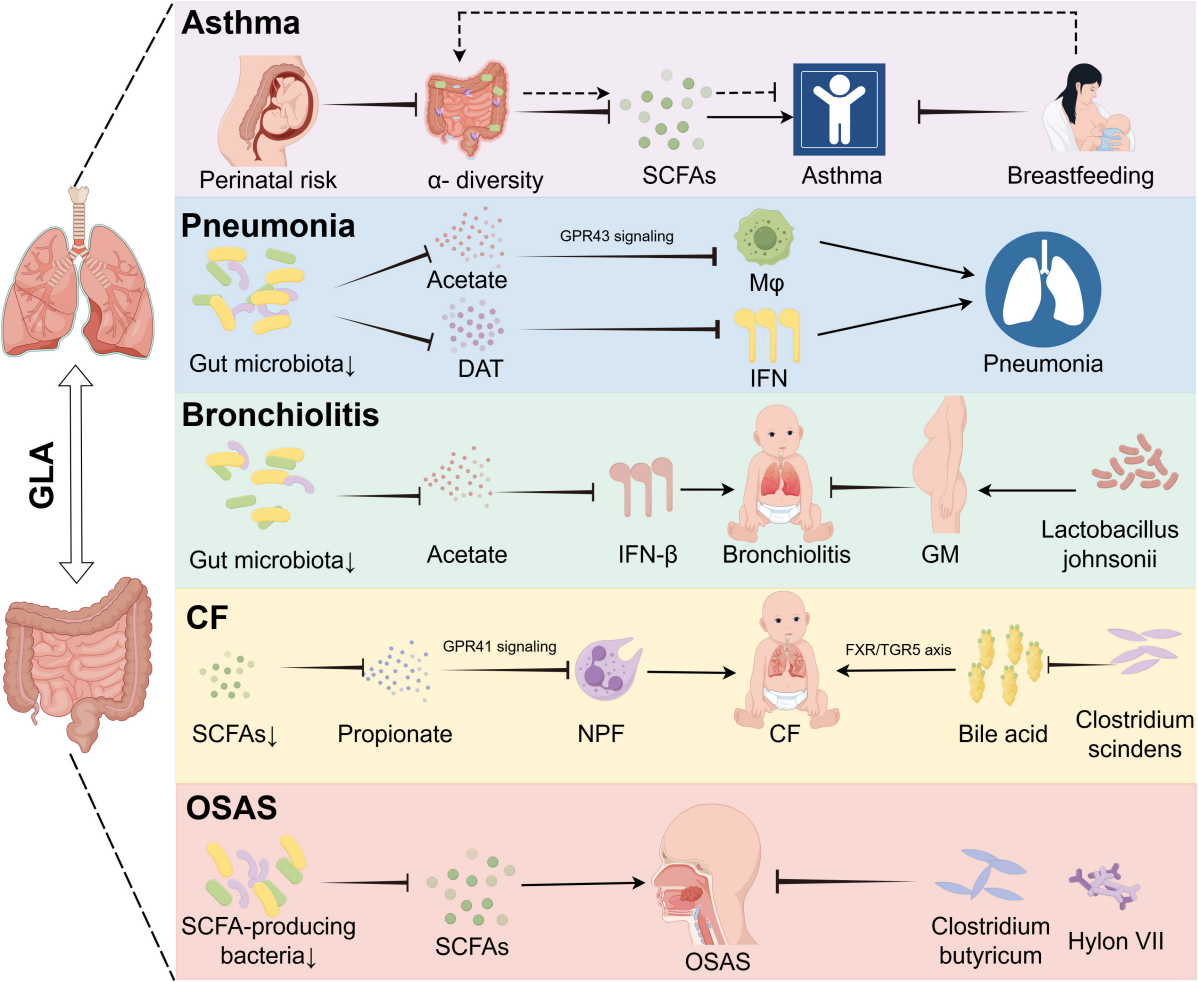
Gut dysbiosis drives disease-specific mechanisms in pediatric pulmonary disorders via metabolic and signaling pathways. The GLA orchestrates disease-specific mechanisms underlying childhood asthma, pneumonia, bronchitis, CF, and OSAS. In children with asthma, perinatal microbiota alterations with reduced alpha diversity are modulated by maternal breastfeeding-associated metabolites. In pneumonia, gut dysbiosis reduces acetate levels, impairing the GPR43-mediated phagocytic function of alveolar macrophages, and deaminotyrosine deficiency compromises IFN-I-dependent anti-influenza immunity. Bronchitis is linked to diminished gut-derived acetate, leading to pulmonary IFN-β reduction whereas prenatal supplementation with Lactobacillus jensenii lowers disease incidence. In CF, Bacteroides-derived propionate deficiency disrupts GPR41 signaling, causing neutrophil phagocytic dysfunction, and Clostridioides difficile depletion exacerbates inflammation via dysregulated bile acid metabolism (FXR/TGR5 axis). Reduced SCFA-producing bacteria contribute to OSAS pathogenesis, which is alleviated by C. butyricum and prebiotics. GLA, gut–lung axis; DAT, desaminotyrosine; GM, gestational mother; GPR43, G protein-coupled receptor 43; IFN-I, type I interferon; Mφ, macrophage; NPF, neutrophil phagocytic function; OSAS, obstructive sleep apnea syndrome; SCFAs, short-chain fatty acids.

Breastfeeding exerts important modulatory effects on asthma-related microbial metabolites (49). Contrary to historical sterility assumptions, human milk contains dynamic microbial communities and prebiotic compounds that shape the infant gut ecology (57, 58). Multivariate analysis has revealed that exclusive breastfeeding ≥4 months is an independent protective factor against asthma development (49), highlighting nutritional–microbial interactions in early-life asthma prevention (**Figure 3**).

### 4.2 Pneumonia

Pneumonia is an infection that inflames the air sacs and other parts of the lungs and is often caused by bacteria, viruses, or other pathogens (59, 60). Pneumonia remains a leading cause of childhood morbidity and mortality, accounting for 14% of deaths in children under 5 years globally, with an estimated 120 million cases annually (61). Research has shown that viral infections, such as influenza, can substantially alter the gut microbiome, even without detectable viral particles in the gastrointestinal tract (62). Compelling evidence links the gut microbiota composition to pneumonia susceptibility and severity (63). Mice with a depleted gut microbiome exhibit increased bacterial spread, heightened inflammation, organ dysfunction, and elevated mortality following *Streptococcus* pneumoniae infection, compared to those with a normal microbiome. Fetal microbiota transplantation (FMT) can restore pulmonary bacterial levels and normalize immune responses in these mice. The gut microbiome modulates metabolic pathways in alveolar macrophages, affecting cellular responsiveness, with macrophages from mice that have a depleted microbiome showing reduced phagocytic activity against *S. pneumoniae* (64) (**Table 1**). Mechanistic studies reveal two key pathways: 1) macrophage priming: gut-derived acetate enhances alveolar macrophage phagocytic capacity against *S.* pneumoniae via GPR43-dependent metabolic reprogramming (65) (**Table 1**); antiviral defense: microbiota-derived desaminotyrosine protects the host from influenza by modulating the type I interferon (IFN-I) response (66) (**Figure 3**).

Pneumonia poses a serious health risk to children worldwide, with high case numbers and mortality rates, emphasizing the need for prevention and better treatment (67). Preterm infants with pneumonia exhibit reduced gut microbiota diversity and disturbances of the gut (68). Children’s underdeveloped immune systems make them vulnerable to severe illness and death due to pneumonia. The gut microbiota, particularly early in life, influences lung immunity and the pneumonia risk, highlighting the importance for health of a balanced gut microbiome (23). Pneumonia can affect the composition of the gut microbiota, primarily leading to a decrease in *Bifidobacterium* (10), *Lactobacillus* (69), and *Clostridium* (70), and causing an increase in *Escherichia coli* (59). Infant gut microbiota diversity and functionality increase during the first years of life, influenced by the delivery mode and feeding type (71). In pneumonia, *Pseudomonas*, *Escherichia/Shigella*, *Streptococcus*, and *Akkermansia* are decreased *Bacillota* are increased, at the phylum level, with specific genera being more or less abundant in cases of pneumonia. Preterm infants with pneumonia exhibit reduced gut microbiota diversity and gut disturbances (68) (**Figure 2**).

The prevalence of pediatric COVID-19 cases ranges from 1% to 13.3% of total reported infections (72). A recent study revealed significantly reduced α-diversity in young patients with COVID-19 (aged <6 months), aligning with prior studies attributing such disparities to the unique infant microbiota, which is shaped by feeding patterns (73). Beyond gut microbiota alterations, intestinal inflammation, microbial translocation, and intestinal barrier dysfunction have also been identified as factors associated with COVID-19 infection and that are potentially attributable to microbiota-driven perturbations (74). The critical role of microbiota has prompted experts to question whether the host microbiota status should be considered prior to vaccine development. Furthermore, the development of oral vaccines and maintenance of microbial homeostasis may facilitate early containment of COVID-19 outbreaks or future pandemics (74).

### 4.3 Bronchiolitis

Respiratory syncytial virus (RSV)-associated bronchiolitis affects 33 million children under age 5 years annually, with 3.2 million requiring hospitalization (75). Emerging data implicate gut microbiota dynamics in disease progression. Cohort studies reveal that infants hospitalized with severe RSV bronchiolitis exhibit gut dysbiosis characterized by *Enterobacteriaceae* overgrowth and *Bifidobacterium* depletion (76). RSV infections are particularly dangerous for infants under age 2 years, leading to high rates of morbidity and mortality. Early infancy is a key period in gut microbiota development that may play a role in bronchiolitis through various mechanisms beyond the Th1/Th2 imbalance theory. Research indicates that acetic acid produced by gut microbes can stimulate lung IFN-β production, enhancing type 1 IFN responses by activating GPR43 and NF-κB (77) (**Table 1**). This activation can modulate the protective effects of acetic acid against RSV, potentially reducing pneumonia symptoms (**Figure 3**).

Current research is investigating intervention strategies for respiratory illnesses, with clinical trials demonstrating that *Bifidobacterium* species stimulated by human milk oligosaccharides contribute to the prevention of subsequent respiratory infections (e.g., bronchitis). This protective mechanism is mediated via *Bifidobacterium*-driven enhancement of acetate production, with elevated acetate levels being mechanistically linked to improved intestinal barrier function (78). Emerging clinical evidence has revealed that maternal supplementation with *Lactobacillus johnsonii* confers transgenerational protection against RSV infection, with offspring exhibiting attenuated airway mucus production and Th2-mediated immune responses. In a murine study, this intervention maintained congruent gut microbiota profiles in both dams and progeny, accompanied by a synchronized reduction in pro-inflammatory metabolites in the maternal plasma, breast milk, and offspring circulation (79). Mechanistically, *Lactobacillus*-driven modulation of maternal microbial ecosystems and associated metabolic reprogramming are positively correlated with enhanced neonatal airway defense mechanisms against RSV pathogenesis (79).

### 4.4 Cystic fibrosis

CF is an inherited disorder transmitted through an autosomal recessive pattern, resulting from mutations in the cystic fibrosis transmembrane conductance regulator (*CFTR*) gene. This defect induces the accumulation of abnormally viscous mucus secretions, which primarily compromise pulmonary and gastrointestinal functions (80). CF affects approximately 70,000 people worldwide (81). Lung infections caused by bacteria such as *Pseudomonas aeruginosa*, *S. aureus*, *Burkholderia cepacia* complex, and *Stenotrophomonas maltophilia* worsen respiratory function and contribute to disease morbidity (19). Research supports the existence of a “GLA” in chronic respiratory diseases such as CF, affecting mucosal immunity across these systems (82, 83). Cross-sectional studies have revealed that pediatric patients with CF exhibit marked gut dysbiosis, characterized by a marked depletion of beneficial taxa including *Bacteroides, Bifidobacterium adolescentis, Faecalibacterium prausnitzii, Actinomyces, Eubacterium, Ruminococcus, Dorea, Akkermansia, Peroxisome-associated Firmicutes and Pantoea* (84, 85), alongside concurrent enrichment of opportunistic pathogens such as *Staphylococci, Streptococci, Veillonella dispar, Propionibacterium*, and *Clostridium difficile* (84) (**Figure 2**).

Mechanistic studies identify two critical pathways linking gut dysbiosis to pulmonary decline. Reduced levels of Bacteroides-derived propionate in SCFA deficiency impair neutrophil phagocytic function via compromised GPR41 signaling (81, 86). Concurrently, diminished *Clostridium scindens* activity disrupts bile acid metabolism by reducing deoxycholic acid production, which exacerbates airway inflammation through dysregulation of the FXR/TGR5 signaling axis (87) (**Figure 3**, **Table 1**).

A prospective cross-sectional study elucidated that children with CF exhibit a dietary pattern characterized by high fat and low fiber, which is associated with intestinal dysbiosis, elevated fecal calprotectin, and respiratory microbial disturbance, collectively leading to impaired lung function and increased lung exacerbation (82). Another study highlighted the therapeutic potential of microbiota-targeted interventions (e.g., probiotics, CFTR modulators) in mitigating systemic and respiratory morbidity in children with CF by restoring the gut microbial balance, enhancing immunomodulation, reversing SCFA deficits, and disrupting pathogenic gut–lung crosstalk, thereby attenuating chronic inflammation and improving pulmonary outcomes (88). These findings highlight the gut microbiome as a modifiable determinant of CF progression, paving the way for microbiota-centric adjuvant therapies.

### 4.5 Obstructive sleep apnea syndrome

OSAS affects 2%–4% of children, with increasing prevalence linked to pediatric obesity and adenotonsillar hypertrophy (89). OSAS is a prevalent chronic respiratory disorder marked by repeated pharyngeal collapse and ventilation disruption during sleep, leading to apnea and sleep disturbances (90). Emerging evidence implicates gut microbiota dysbiosis as both a contributor and consequence of OSAS pathophysiology (91, 92). Cross-sectional studies demonstrate that children with moderate-to-severe OSAS exhibit gut microbiome alterations characterized by an increase in *Clostridia* and *Ruminococcus* species, coupled with depletion of SCFA-producing bacteria such as *Faecalibacterium* and other taxa associated with SCFA synthesis (93, 94) (**Figure 2**).

In one study, researchers hypothesized that diminished intestinal SCFAs underlie hypertension pathogenesis in OSA. OSA significantly elevated systolic blood pressure after 7 and 14 days, an effect abolished by administration of the probiotic *Clostridium butyricum* or the prebiotic Hylon VII. 16S rRNA sequencing revealed significant enrichment of SCFA-producing bacterial taxa following C. butyricum or Hylon VII treatment. OSA exposure reduced cecal acetate concentrations by 48%, which was prevented by both interventions. Furthermore, C. butyricum and Hylon VII attenuated OSA-induced gut dysbiosis, goblet cell depletion, mucus layer thinning, and cerebral microglial activation (95) (**Figure 3**, **Table 1**). A systematic review and meta-analysis indicated that OSA is associated with intestinal barrier dysfunction. Furthermore, the severity of OSA appears to be associated with elevated levels of biomarkers of intestinal barrier dysfunction (96). These preclinical findings suggest that microbiota modulation may serve as a potential adjuvant strategy for OSAS management, although clinical validation in pediatric populations is warranted.

## 5 Clinical importance of GLA research

### 5.1 GLA as a biomarker for pediatric pulmonary disease diagnosis

The gut microbiota and its metabolites are increasingly recognized as potential biomarkers for pediatric pulmonary diseases. Omics approaches have been used to identify microbial signatures associated with disease states, including an elevated *Firmicutes/Bacteroidetes* ratio indicative of dysbiosis in respiratory conditions (15, 97). In COVID-19, the gut microbiota composition correlates with disease severity and immune dysregulation, characterized by depleted SCFA-producing taxa and impaired L-isoleucine biosynthesis (98, 99) (**Table 1**). Experimental models have further demonstrated metabolic disturbances, such as increased serum lactate and reduced acetate levels in pulmonary hypertension (97). Although these findings underscore the diagnostic potential of microbiota profiling, current evidence remains fragmented, necessitating large-scale longitudinal studies to validate microbial and metabolic biomarkers across diverse pediatric populations (15).

### 5.2 Potential application of the GLA in treating pediatric pulmonary diseases

Therapeutic strategies targeting the GLA show promise in modulating immune responses and improving clinical outcomes. Probiotics, such as *Lactobacillus plantarum*, enhance IFN-I production and reduce viral loads in influenza models whereas *Clostridium orbiscindens*-derived desaminotyrosine strengthens antiviral defenses (62, 69). SCFAs exert systemic anti-inflammatory effects by promoting Treg differentiation (via HDAC inhibition) and suppressing Th2/Th17 polarization (through GPR41/43 signaling), with fecal propionate levels inversely correlated with asthma risk in infants (39, 40). Omega-3 polyunsaturated fatty acids (PUFAs) such as DHA (docosahexaenoic acid) and EPA (eicosapentaenoic acid) can regulate immune cell functions, reduce inflammatory responses, and enhance antiviral immune reactions, thereby playing a protective role in pediatric pulmonary diseases. Research has found that prenatal supplementation with omega-3 PUFAs can influence the airway microbiota of infants, reducing the risk of respiratory infections (100). Despite these advances, standardized protocols for strain selection, dosing, and long-term safety monitoring remain critical challenges in clinical translation.

## 6 Challenges and future directions in GLA research

### 6.1 Current challenges in GLA research for pediatric respiratory diseases

Despite growing interest in the GLA and its role in pediatric respiratory disorders, critical methodological and translational gaps hinder progress. A predominant reliance on cross-sectional studies limits causal inference because observed associations—such as the reduced gut microbiota diversity in asthma—cannot be used to distinguish whether dysbiosis drives disease or vice versa. Large-scale longitudinal cohorts tracking microbiota dynamics from early life to disease onset are essential to resolve such ambiguity. Furthermore, small sample sizes (<100 participants) and geographical biases (e.g., overrepresentation of European populations) reduce generalizability, and inconsistent sample handling protocols introduce technical variability that obscures biological signals.

The complexity of gut microbiota data presents another layer of difficulty. Integrating taxonomic, metabolic, and functional profiles requires advanced bioinformatics capabilities that are often unavailable to smaller research teams. Compounding this issue, key confounders such as diet are insufficiently addressed. Most studies focus narrowly on the short-term effects of isolated nutrients, overlooking the cumulative impact of dietary patterns and individualized eating behaviors. Genetic predispositions and environmental exposures (e.g., air pollution, antibiotic use) further interact with microbiota in ways that remain poorly characterized, highlighting the need for multifactorial models.

Translating preclinical findings to clinical applications involves additional hurdles. Although animal studies have elucidated mechanisms such as SCFA-mediated immune modulation, they fail to replicate the complexity of human pediatric immune development and environmental exposures. Clinical trials must navigate inherent challenges including stringent participant matching for age, feeding practices, and comorbidities; prolonged follow-up (≥5 years) to assess outcomes in slowly progressing diseases; and unresolved safety concerns regarding probiotics or microbiota-targeted therapies in children.

Finally, the dynamic nature of the gut microbiota during childhood complicates research and intervention design. Most studies capture static snapshots, ignoring developmental trajectories and cumulative microbial effects on disease. Pronounced inter-individual variability, shaped by gene–environment–diet interactions, further complicates the development of personalized approaches. Addressing these limitations requires multinational longitudinal cohorts, multi-omics integration driven by artificial intelligence, and pediatric-specific therapeutic frameworks to advance GLA research from correlation to causation and clinical utility.

### 6.2 Development and clinical trials of novel treatments

#### 6.2.1 Fecal microbiota transplantation

**Figure 4:**
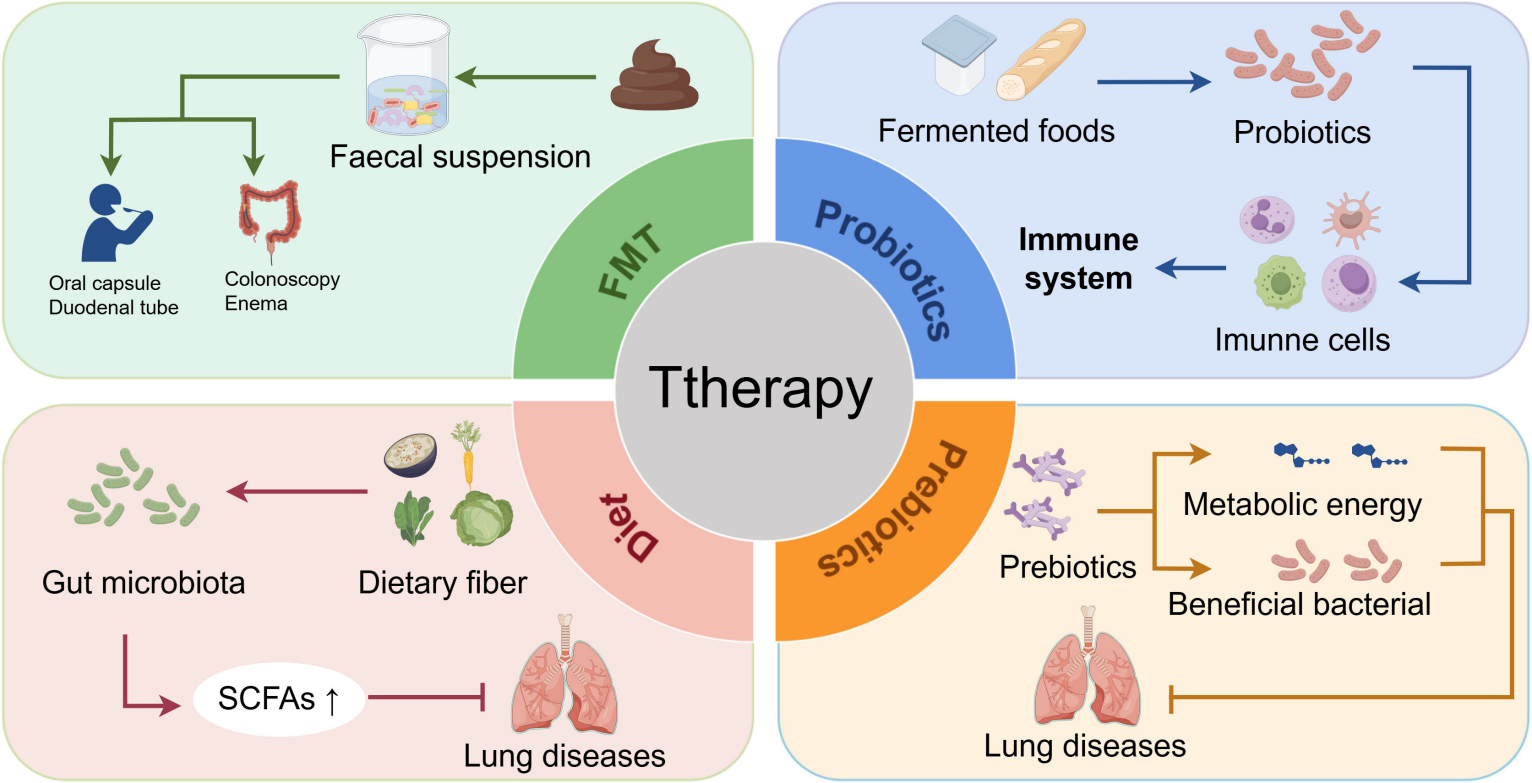
Therapeutic strategies targeting the GLA for pediatric pulmonary diseases: mechanisms and clinical interventions. Targeting the GLA offers a novel approach to managing childhood pulmonary diseases through gut microbiota modulation. FMT restores microbial homeostasis by administering healthy donor fecal suspensions via oral capsules, duodenal tubes, or colonoscopy/enema delivery. Probiotics alleviate respiratory symptoms via immunomodulation; prebiotics promote the growth of beneficial bacteria and provide metabolic substrates, thereby reducing pulmonary disease risk. Dietary interventions reshape gut microbiota composition, enhance SCFA production, and mitigate disease progression (e.g., pneumonia). Current evidence indicates that probiotics are more effective against eczema and allergic rhinitis than against asthma, highlighting the need to optimize strain selection, dosage, and treatment duration to improve GLA-targeted therapeutic precision. FMT, fecal microbiota transplantation; GLA, gut–lung axis; SCFAs, short-chain fatty acids.

FMT is a treatment method in which a fecal suspension obtained from a healthy donor is transferred to the patient’s digestive tract to restore the normal microbial composition and function of the intestinal tract (101, 102). FMT can be delivered through the upper GI route via a duodenal tube or capsules taken orally (103) or through the lower GI route via colonoscopy or enema (104) (**Figure 4**). FMT may have applications in the treatment of many diseases, such as asthma, and has been used to treat a variety of gastrointestinal and non-gastrointestinal disorders (105).

#### 6.2.2 Probiotics

Interest in probiotics has grown substantially owing to their complex mechanisms, which are strain- and compound-specific (106). The mechanisms by which probiotics operate are intricate and varied, typically depending on the specific strain and compound (107). Probiotics can alter the gut microenvironment, compete with harmful bacteria, and suppress pathogens using antimicrobial substances, thereby influencing gut health in nuanced ways (59). Emerging strategies targeting OSA-related gut dysbiosis using prebiotics, probiotics, and SCFAs are being considered for lung disease management and represent a promising therapeutic avenue (91, 108). Probiotics are present in fermented foods and supplements as scientifically validated beneficial strains and exhibit bidirectional immunomodulatory effects on the host, being capable of inducing pro-inflammatory responses while eliciting anti-inflammatory reactions (109). Under immunostimulatory conditions, macrophages, DCs, neutrophils, and natural killer cells in the intestinal mucosa participate in immune responses through enhanced phagocytic activity, inflammatory cytokine release, and Th1/Th17 polarization (15). Concurrently, probiotics activate innate immune responses and cytokines secreted by T cells to stimulate lamina propria-associated immune cells. This immunomodulatory activity is specifically characterized by inducing the production of immunoglobulin A antibody, increased population density, and functional enhancement of macrophages and DCs within the lamina propria, thereby sustaining their functional reinforcement in the small intestine (110) (**Figure 4**). A placebo-controlled, double-blind, randomized study investigated the effects of a probiotic mixture containing *Bifidobacterium longum BB536*, *Bifidobacterium infantis M-63*, and *Bifidobacterium breve M-16V* in 40 children with seasonal allergic rhinitis and intermittent asthma over a 4-week intervention period. Children receiving the probiotic supplementation demonstrated significant reductions in respiratory symptoms and improved quality of life whereas the placebo group exhibited symptom exacerbation and quality of life deterioration (111). A meta-analysis suggested that Lactobacillus rhamnosus GG (LGG) supplementation, especially postnatally, may prevent asthma (112); however, a database review indicated that probiotics are more effective for eczema and allergic rhinitis than for asthma prevention or treatment (113). Health organizations emphasize the need for further research on probiotic efficacy in asthma prevention because studies may not have used the appropriate strain, dosage, timing, duration, or population (114). Future research will focus on the duration, administration, dosage, and follow-up period for specific probiotic strains (115).

#### 6.2.3 Prebiotics

According to the updated scientific definition established by the International Scientific Association for Probiotics and Prebiotics in 2017, prebiotics are defined as a substrate that is selectively used by host microorganisms to confer a health benefit (116). These compounds exert their effects primarily via two mechanisms: 1) by modulating the gut microbiota composition through the selective promotion of beneficial bacterial growth and provision of metabolic energy (117) (**Figure 4**); and 2) by enhancing intestinal barrier function and stimulating the production of beneficial metabolites, thereby inducing multifaceted physiological regulation in the host (118). Given their potential immunomodulatory properties and mechanistic actions, a theoretical basis exists for prebiotics to potentially reduce the risk of COVID-19 infection or mitigate its clinical symptoms (119). However, this hypothesis requires rigorous validation in further experimental and clinical investigations (120). A review of 2,419 pediatric participants analyzed asthma exacerbations, pulmonary function, and immune modulation. Studies that have focused on Lactobacillus/Bifidobacterium (10 randomized controlled trials [RCTs]), bacterial lysates (6 RCTs), and synbiotics (2 RCTs) represent a notable paucity of prebiotic research (121).

#### 6.2.4 Diet

Studies have shown that factors such as diet, genetics, and age affect diversity of the gut microbiota, with diet being a key modifiable factor for treating dysbiosis (27, 122). Clinical and preclinical data highlight how dietary changes can rapidly alter the gut microbiota composition, such as shifts occurring within 24 hours of switching from an animal-based and to a plant-based diet (123, 124). Dietary fiber intake boosts bacterial metabolites, particularly SCFAs (41). Dietary patterns influence the β diversity of the gut microbiota without affecting its α diversity, which varies among individuals consuming an animal-based diet (125, 126). High-calorie diets can worsen LPS-induced pneumonia by disrupting the gut microbiota balance and Th17/Treg cell ratios (127). Future prevention and therapy may involve dietary pattern modifications, such as reducing specific nutrients or adopting lifestyle changes to address physical inactivity and obesity (**Figure 4**).

Probiotics and dietary changes aim to foster a balanced gut microbiota, which is beneficial for managing respiratory conditions and offering a safer alternative to traditional pharmacological treatments, especially in chronic disease management (3). These strategies collectively bridge mechanistic insights and clinical translation to reshape pediatric respiratory disease management.

### 6.3 Future research directions

The role of the gut microbiome in pediatric respiratory diseases is an emerging field with implications for new therapeutic strategies. Future research should focus on the GLA, particularly the impact of microbial metabolites on immune modulation. Multi-omics approaches will help deepen understanding regarding the complex interactions between the gut microbiota and children’s respiratory health. A key objective is to identify microbial signatures and metabolites that serve as biomarkers for early diagnosis and prognosis of diseases such as asthma and pneumonia, enabling personalized medicine based on individual microbiome profiles. It is also crucial to explore the role of the gut microbiome in responses to treatments such as FMT and probiotics/prebiotics, as well as to design clinical trials assessing their efficacy and safety. Research should also investigate the microbiome’s influence on vaccine responses and immunotherapies for respiratory diseases, potentially leading to microbe-based adjuvants that boost vaccine efficacy. The impact of diet on the gut microbiome and lung health in children merits further study, with a focus on dietary interventions to improve respiratory outcomes. Finally, translating preclinical findings into clinical practice is essential. Future research should connect laboratory discoveries with clinical applications, implementing evidence-based interventions that leverage the GLA to prevent and treat pediatric respiratory diseases, with the aim to reduce the impact of these diseases on children’s health and quality of life globally.

## 7 Conclusions

This review synthesizes critical evidence on the MGLA as a central regulator of pediatric respiratory health. Early-life gut microbiota perturbations—driven by birth mode, antibiotics, and diet—disrupt immune programming via metabolite signaling (e.g., SCFAs), epigenetic modulation, and pathogen exclusion, predisposing to asthma, pneumonia, and bronchiolitis. Multi-omics integration has revealed evolutionarily conserved microbiota–immune network architectures across disease phenotypes, with mechanistic prioritization identifying Faecalibacterium-derived SCFA-mediated Treg modulation as therapeutically actionable biological circuitry. To systematically characterize the methodological framework underpinning these findings, we constructed a comprehensive reference table (**Table 1**) detailing the multi-omics platforms, analytical pipelines, and data integration strategies used, thereby providing explicit technical documentation to enhance methodological reproducibility and cross-study interoperability. Clinically, microbiome-modulating strategies—including precision probiotics, FMT, and dietary SCFA boosters—demonstrate efficacy in respiratory diseases such as asthma and pneumonia. For OSAS, the current evidence is primarily derived from preclinical models, necessitating clinical trials to establish therapeutic efficacy. Future research must prioritize longitudinal birth cohorts to map developmental windows of microbiota–immune crosstalk, coupled with mechanistic studies dissecting microbial vesicles and mobile immune cell trafficking. Standardizing microbiota therapeutics and leveraging AI-driven biomarker panels will accelerate translation from bench to bedside, ultimately enabling the personalized management of childhood respiratory diseases.
